# The Interpretation of Dreams

**Published:** 1914-03

**Authors:** 


### Reviews of Books.

**The Interpretation of Dreams.** By Prof. Dr. SIGMUND FREUD. Authorised Translation of Third Edition by A. BRILL, M.D. Pp. xiii, 510. London: George Allen & Co. Ltd. 1913. 15s. net.

This new work of Freud shows further proof of his remarkable ability in psychological analysis, and has added greatly to our knowledge of the dream, its exciting and determining factors, meaning and relationships. We think, however, that the author pushes his subtilty in analysis and suggestion in questioning too far and may assume too much from the result. The inference of a sexual origin in nearly all cases we cannot think warranted, although, no doubt, specimens of this kind gravitate towards the specialist who invites them. The book is too long and complicated to allow of its many conclusions being summarised in the short space here available. The main assumption is that desires and apprehensions which have been suppressed find an outlet in dreams, but one more or less modified by the resistance of the "censor," which does not lose its power even during slumber. Hence they appear in a symbolic form which requires certain known methods of interpretation to be employed for its recognition. Thus, dreams may have a double meaning, a manifest, which is immediately evident, and a latent which is only disclosed by analysis. The latter process consists of the employment of known methods of association-revival such as Freud and Jung have perfected. The analysis of dreams is made for the same purpose and much on the same lines as psycho-analysis in the waking subject. In this work will be found certain definite rules upon which dreams are constructed, manifested and correctly interpreted.

